# Parental experiences in neonatal intensive care unit in Ethiopia: a phenomenological study 

**DOI:** 10.1080/07853890.2021.2004320

**Published:** 2022-01-08

**Authors:** Endalkachew Worku Mengesha, Desalegne Amare, Likawunt Samuel Asfaw, Mulugeta Tesfa, Mitiku B. Debela, Fentie Ambaw Getahun

**Affiliations:** a School of Public Health, College of Medicine and Health Sciences, Bahir Dar University, Bahir Dar, Ethiopia; b School of Health Science, College of Medicine and Health Sciences, Bahir Dar University, Bahir Dar, Ethiopia; c Department of Epidemiology, Hosanna Health Science College, Balie, Ethiopia; d Department of Epidemiology, College of Health Sciences, Debre Markos University, Debre Markos, Ethiopia; e School of Public Health, College of Medicine and Health Sciences, Maddawalabu University, Balie, Ethiopia

**Keywords:** Parents, experiences, infants, neonate, neonatal intensive care unit, phenomenology study

## Abstract

**Introduction:**

Neonatal intensive care unit is important to save the lives of a sick neonate; however, parents are challenged by several stressful conditions during their stay. Therefore, this study aimed to explore the lived experiences of parents in neonatal intensive care units in Ethiopia.

**Methods:**

We used a phenomenological study design. The data were collected using an in-depth interview method from purposively selected parents. In addition, we followed a thematic analysis approach and used Open Code Software Version 4.02 to process the data.

**Results:**

In this study, 18 parents were interviewed. The researchers have identified six themes. Parents complained of psychological problems like anxiety, stress, worries, hopelessness, and a state of confusion. In addition, anger, crying, sadness, frustration, dissatisfaction, regret, disappointment, feeling bad, self-blaming, nervousness, disturbance, and lack of self-control were major emotional problems raised by the parents. Parents expressed that health care providers showed indiscipline, lack of commitment, and uncooperative behaviour. Likewise, shortage of medicines, money, and limited time to visit their neonates were the other concerns of many parents. At the same time, parents were provided minimal information and limited cooperation from health care providers.

**Conclusion:**

Parents whose infants admitted to the NICU were suffered from various psychological and emotional problems. Researchers recommend that health care providers should be supported parents with psycho-emotional problems, strengthen parents–healthcare workers' interaction, and scale up neonatal intensive care unit services to the primary health care centres.

**KEY MESSAGES**
- Parents whose infants admitted to the NICU were suffered from psychological and emotional problems.
- Poor NICU environment, shortage of equipment, long hospital stay, the presence of pandemic COVID-19, and lack of parental involvement in the care were identified barriers that affected parents' stay.

## Introduction

The neonatal intensive care unit (NICU) is a unit that provides day and night care for sick or preterm infants [1]. The NICU has specialist medical staff and equipment to care for premature and sick newborn babies. This part of the hospital is sometimes called the intensive care nursery [1,2]. The creation of special care units for infants was sparked by the realisation that heat, humidity, and a steady supply of oxygen could increase the survival rates of sickly babies, meaning that hospitals could intervene to help babies live as opposed to just sending them home [3]. Parental stress related to NICU admission is a worldwide healthcare issue [4]. According to the American Academy of Paediatrics, nearly 5% of all newborns require an intensive care unit stay. Another 15–20% of all newborns require specialty services [1].

In Ethiopia, a well-structured neonatal intensive care unit was expanded in public hospitals since 2013, and to produce the required adequate numbers of health units to staff the NICU, training was given to health care providers on various aspects of specialised neonatal intensive care provision [2]. The national-level prevalence of preterm birth in Ethiopia was high. A systematic review and meta-analysis showed that the overall pooled prevalence of preterm birth in Ethiopia was 10.48% [5].

Parents experienced many challenges in the NICU due to neonates’ health status, inadequate information, and lack of support from healthcare providers (HCPs) [6–9]. They also suffered from socio-economic, psychological, emotional, and physical health problems [10].

NICU parents experienced many stressful psycho-emotional conditions like anxiety, worries, shock, fear, dissatisfaction, anger, and post-traumatic stress during admission and after discharge [7,11–13]. Additionally, parents explained that prematurity, low birth weight, the severity of illness, and poor prognosis of a child were common causes for stress. They were stressed when the HCPs told about worsened medical conditions such as irregular breathing patterns and attached medical devices to their baby [14–18]. Other studies showed that poor parents-HCPs interaction [19–21], limited counselling and inconsistent information obtained from HCPs led to parental stress [9,21–23]. However, parents report feeling less stressed as they receive HCP support [24].

Parents had the main role in neonatal care in the NICU [13,25] and need supportive measures to relieve stressful conditions [26,27]. Parents gave witness that HCPs tried to save their severely ill neonates [28,29] but, their support was minimal [17,30]. Understanding the emotion of parents is essential to improve neonatal health conditions [10,26]. This helps to design stress-relieving measures like counselling, education, and support [31].

Parents whose neonates were subjected to severe medical problems experienced the feeling of exclusion and lack of belongingness to the NICU [11,13,32,33]. Likewise, parents felt that the care provided in NICU is compromised due to HCPs' work overload, disease-oriented nursing care, longer hospital stay, shortage of healthcare providers, lack of beds and medical equipment [34,35]. However, parents had a sense of encouragement, empowerment, and empathy when the HCPs understand their feelings [13,36].

Although parental experiences in the NICU was explored in different countries [7,10,29,36–38], there are variations in experiences, accessibility of health facilities, parent-HCPs interactions and parental involvement in the care due to socio-cultural differences, nurses’ working culture differences and variation in the hospital setting [39–42]. It is not also well explored in Ethiopia. The information generated from this study is essential to the hospital to improve the services by facilitating NICU resources. In addition, it would help programmers to formulate strategies targeting the NICU and scale up the service to the periphery level. Therefore, this study aimed to explore the lived experiences of parents whose premature infants were admitted into a NICU.

## Materials and methods

### Study area and period

This study was conducted in one of the hospitals in Ethiopia from August 1 to 15, 2020. The hospital was established in 1961 and currently provides services for about seven million people. The hospital has seven wards including the NICU. It also has 859 HCPs (133 physicians, 438 nurses, and 56 midwives). Specifically; six physicians and 31 nurses were working in the NICU and there were 83 beds with supportive machines. The NICU service was established as a separate department in February 2015. Currently, the unit comprises Kangaroo Mother Care (KMC) with 8 beds, maternal side with 33 beds, term with 14 cots, preterm room with 20 cots, and septic room with 8 cots. The unit is equipped with 11 radiant warmer machines, 2 heaters, an average of 20 oxygen cylinders per 15 days, four perfusion machines, four electrical continuous positive airway pressure(CPAP), 10 non-electrical CPAP, and two mechanical ventilators. Besides, bubble CPAP prepared using locally available materials like ringer lactate bag, tap water, and oxygen cylinder was used. However, there is no automated CPAP and mechanical ventilator.

According to 2019, six months reports showed that 1296 neonates were admitted to the NICU. The average monthly admissions of infants were about 180. All newborn infants are admitted at any age of gestations. In NICU, all the services are free of charge.

The NICU ward was crowded and there was no space for family attendants and visitors to take a rest, and there were no waiting rooms and chairs, most visitors and attendants sit on maternal beds. On the neonatal side, more than six babies have admitted within one room and more than two babies were under one radiator warmer or phototherapy. On the maternal side, more than ten mothers were admitted in one room. The building lacks a special provision for disabled parents and the elevators were not functioning properly at the time of the study.

### Study design

We used a descriptive phenomenology approach to explore the lived experiences of parents when their infants were admitted to NICU. The purpose of this phenomenological study was to explore how parents experiences and understand the service provided to their infants. To assess parents' lived experiences, the phenomenology study design is more powerful than other designs and well suited for exploring challenging events of parents in the hospital stay.

### Selection of participants and recruitment

During the data collection period, more than 30 neonates were admitted to the NICU. A total of 56 parents were eligible to participate in the study. Of these, 19 parents were approached to interview, but a total of 18 parents were interviewed (nine mothers and nine fathers were interviewed). One parent was not volunteer for interviewing, thus her husband was invited and interviewed. Only one parent either mother or father reporting for one infant. Parents were eligible to participate in the study regardless of the mode of delivery and health status of the infants. To recruit the NICU parents, first, we registered details of different medical characteristics of admitted neonates such as low birth weight, prematurity, sepsis, perinatal asphyxia, meconium aspiration syndrome, respiratory distress syndrome, and jaundice. Parents with a minimum of 72 h stay in the hospital were purposively selected to get complete and adequate information in their stay.

### Data collection

Data were collected by researchers who have plenty of experiences using a semi-structured interview guide. Parents were interviewed in the nursing duty rooms using an audio recorder. The research team was developed a semi‐structured in-depth interview (IDI) guide. The main IDI guiding questions included in this study were mentioned as follows: What is your feeling when the healthcare providers informed you that the neonate has to be admitted to the NICU? What do you feel when you observe your baby is critically ill? How do you feel when the medical devices are attached to your newborn child? How do you explain your involvement in providing care to your baby in the NICU? What are the difficulties you faced after the newborn infants were admitted to the NICU? How do you explain the supports given by HCPs and the hospital? How do you explain the services received in the NICU? What is your suggestion for the improvement of NICU services? And how do you feel about your stay here in the NICU setting? The IDI guide was translated into the local language Amharic. Before the actual data collection period time, a pre-test was done at a nearby hospital by research teams, and a mild amendment was done on the language difficulty of the interview guide. Parents were interviewed in a quiet room adjacent to a maternal side room. The English version of the guiding questions has been included in the supplementary file (S1_File). The time length of each interview was taken from 30 to 60 min with a mean time of 45 min. Data collection was continued until adequate, complete, and little new information came from the interviewees.

### Trustworthiness

Trustworthiness was verified to address the dimension of credibility, transferability, dependability, and conformability [43]. The credibility of the study was ensured by having prolonged engagement in the field and interaction with parents to obtain in-depth data. Hence, we made verification of the transcribed and translated data by inviting experienced researchers and by repeated engagement of authors in the transcription, translation, and coding. Credibility can also be operationalised through the process of member checking to test the findings and interpretations with the participants' voices. The member checking process was critical to check an accurate representation of the participants' voices. Therefore, we read the key findings and analysis of two randomly selected interviews to them and they confirmed that their voice was articulated in the right way and agreed with the interpretation. In addition, a thick description of the data was done to increase the transferability of the study. Generally, transcription; translation, and interpretation of data were done using scientific procedures. Finally, conclusions were drawn from the data.

### Data analysis

The recorded interview was transcribed verbatim and translated into the English language for the subsequent analysis. The consistency of the transcribed data was checked by listening and reading repeatedly. When there were variations in translation, the research teams discussed and explained the results after the consensus was reached. Data coding was done sentence by sentence to create themes that have similar ideas and thematic analysis was used to describe the data obtained from interviews. Open Code Software Version 4.02 was used to analyse the data.

### Ethical consideration

Ethical approval was obtained from the institutional review board (IRB) of the College of Medicine and Health Sciences, Bahir Dar University with protocol number 00255/2020. A permission letter was also written to the hospital to precede the data collection procedure and permission was obtained from the hospital administrator. The objective of this research was clearly stated to the study participants. Since the data collection was non-invasive, we used oral informed consent from each parent before data collection has begun. The data were collected after study participants affirmed their voluntariness to participate in the study. To maintain the confidentiality of information, we used anonymous codes such as interview parent 1(P1), participant 2 (P2), etc.

## Results

### Socio-demographic characteristics

In this study, 18 parents of a premature infant admitted to NICU were interviewed. All of the parents were Orthodox Christians while 17 of them were married and eight parents were farmers. Seven parents had secondary and above educational level and 12 parents were from a rural area. Of the total parents, four of them travelled more than 100 kilometres to arrive at FHCSH (Table 1).

**Table 1.** Socio-demographic characteristics of interviewed parents in Ethiopia, 2020.

Variables	Categories	Frequency
Mean(46) age(years)		30.6(±7.3)
Age range(years)		20–41
Sex	Male	9
	Female	9
Educational status	Cannot read and write	5
	Can read and write	2
	Primary	4
	Secondary and above	7
Residence	Urban	6
	Rural	12
Marital status	Married	17
	Unmarried	1
Occupational status	Housewife	4
	Government employee	8
	Priest	3
	Student	2
		1
Distance in Km	<15	4
	15–49	5
	50–100	5
	>100	4

#### Infants’ characteristics

A total of 18 parents with 20 infants or newborn babies were involved in the study, four of whom had twins and 16 parents had single babies. Thirteen infants are female and 7 were male infants. Of these, 18 were born prematurely and had various problems and two infants were term. Of these, seven infants were admitted for very low birth weight, four infants with Jaundice, two infants were developed perinatal asphyxia and three infants were admitted with meconium aspiration syndrome. The remaining four infants were admitted with sepsis.

#### Description of parental experiences

The data have shown that parents whose neonates were admitted into the NICU were exhausted, and bored due to prolonged stay, and financially insecure to fulfil their daily living expenses. They could not access a space to take physical rest, and clean water to keep their hygiene. Additionally, they were suffered from longer separation from their neonates due to limited visiting time that is regulated by the hospital. They were uncomfortable with the care given to their neonates because they were restricted to engage the care of neonates. Regarding the health condition of the neonates, parents have a fear that their neonates might die. They are also concerned with seasonal occasions such as COVID-19 transmission that might compromise the care of their neonates. At the same time, parents who are farmers were emotionally unstable for they came to the hospital during the farming season, and due to this reason, they were highly interested to return to their home.

### Themes

In this study, six main themes were identified. These include socio-economic factors, health facility-related factors, parents-healthcare providers' communication, maternal and child health-related factors, psycho-emotional factors, and current occasions (Table 2).

**Table 2.** Summary of Themes, sub-themes, and codes of parental experiences in Ethiopia, 2020.

Themes	Theme-I	Theme-II	Theme-III	Theme -IV	Theme-V	Theme-VI
	Socio-economic factors	Psycho-emotional factors	Parents-health care providers communication	Health facility-related factors	Maternal and child health factors	Current occasions
Sub-themes	Social factors Economic factors	Psychological factors Emotional factors	Communication Parental involvement	Resource related Health workers related factor	Maternal health condition Child health condition	Seasonal factors COVID-19
Codes	Long-distance Shortage of money	Confusion Hopeful Hopelessness Stress Worries Angry Complain Cry Sadness Disappointment Dissatisfaction Fear Bad felling Happiness Self-control Nervousness Rerate Self-blaming	Communication Health information HCPs cooperation Commitment HCPs Support	Absence of HCPs Availability of foods Comfortable with services Eger to discharge HCPs Delay Unavailability of medicines Lack of space Lack of water Limited visiting time Long stay No free bed for admission Uncomfortable setting	Improvement child health Poor suck reflex Maternal illness	Fear of COVID-19 Farming season

#### Theme I: socio-economic factors

Health service delivery in the NICU was free from charges for both maternal and newborn services and many of them the parents were happy with this free service. However, since parents stayed in the hospital for a prolonged time and increased their expenditure, suffered from a shortage of money. “Since I was not expecting that we will stay for several days, now I am running out of money. Hence, I faced a problem in shortage of money”. [P5]

According to this study, travelling a long distance from home to health facilities affected parental experiences in NICU. The distance from home to health facilities that had NICU service ranges from 5 to 240 km.

*“I felt a fluid [flow of amniotic fluid from the uterus] three days before my delivery. When I went to the nearby health center on next day, they [health care providers] told me that it [amniotic fluid] started to clear the canal for delivery. However, they [HCP from the health center] referred me on the third day to the hospital and I delivered my baby at 12:00 am in the hospital. After delivery, they took the neonate into the NICU, because the neonate was weak due to staying in labor for several hours”.[P3]*

#### Theme II: psycho-emotional factors

##### Psychological factors

In this study, the majority of the parents experienced anxiety, stress, worry, hopelessness, and confusion. *“I was coming to this health facility by referring from the health center. My babies [twins] were born at seven months [gestational age] and I have a little bit of trouble. I am worrying if I will go to my home with the loss of my babies”.[P9]*

Additionally, the respondent stated that

*“…my husband left me, and he is not called me. I am worried about what would happen to him. I am also frustrated that something may be wrong with my husband when he goes back home. So, I am feeling hopeless since I am not sure whether my husband arrives safely or something happened to him”. [P9]*

Another respondent described her experience as *“…when I saw my child assisted by machines [nasal cannula and other materials], I feel hopeless”.[P6]*

Another respondent said that: *"I am stressed. The only thing that I can do is giving everything to God to help us".[P3]*

Similarly, one parent described his experience as*: “The presence of my neonate in NICU has made my social life more difficult. My family has become too much confused. In addition, as the health status of my wife is not good, I am worrying too much about her”.[P5]*

However, some parents were happy and hopeful when the health condition of their neonates has improved. For instance, the parent described this as: "*I am praying. As I expect, my neonate health status has improved from day to day and able to sucks breast. I am really happy and hopeful”.[P10]*

##### Emotional factors

This study revealed that emotional factors like being angry, crying, sadness, frustration, dissatisfaction, happiness, regret, compliant, disappointment, bad feeling, self-blaming, nervousness, disturbance, and lack of self-control play a great influence on the parental experiences in the NICU.

The majority of the parents whose neonate health condition had gotten worsen expressed their extreme sadness, disappointment, bad feeling, and anger. For instance, one parent stated: "*I was immersed in deep bad feeling, anger, and sadness. I was also disappointed, disturbed and I was crying a lot”.[P9]*

Besides this, parents experienced that: *“… I was nervous and I experienced lack of self-control, especially, when the health care providers told me that the neonate is seriously ill”.[P14]*

In addition, one parent said: *“I am feeling sad and blaming myself because I had to give my breastfeed as soon as my baby was born.* She also added: *“… the current health condition of my neonate has deteriorated and has made me feel bad. In general, it is a frustrating and dissatisfying moment for me”.[P3]*

Moreover, another parent also described this as: *“It is a difficult moment for me. I am feeling bad for my neonate is critically sick. But, other parents have given me hope that the neonate’s health status will be improved. To be honest, I did not expect the neonate would have such kind of health problem because of neonate’s normal gestational age”. [P2]*

#### Theme III: parents-health care providers communication

##### Communication

This study showed that parents-HCPs communication has influenced parental experiences in NICU. Parents expressed that they have good communication with HCPs during their stay in the NICU. A parent described that:

*“When I need information about the progress of my neonate’s health condition, I can talk directly with the HCPs. I am satisfied with the interaction that I had with them. The HCPs are good in providing health information”.[P11]*

In addition, the parent said: "*I am very satisfied with the HCPs’ communication and cooperation”.[P12].* Another parent also explained that: *“The health team is very kind and cooperative. We have good communication with the health teams. … like wisdom is given for King Solomon, I wish them that God may give long life and may make grow in their profession”.[P1]*

##### Parental involvement

This study indicated that parental involvement has contributed to the improvement of critically sick neonates. A parent described this as: "*I am keeping the medical equipment not to detach from the neonate’s body. I have also other roles like breastfeeding, keeping personal hygiene".[P15]*. Another respondent said that *“My role in the NICU is not beyond facilitating medicines and laboratory requests”. [P10]*

#### Theme IV: health facility-related factors

##### Resource related

This study showed that parents experienced unavailability of some medicines, shortage of water for toilet and hygiene, lack of spaces to take rest, and limited time to visit their neonates. All of the parents described that the time to visit their infants was limited. According to their experiences, they visited their neonates only as per the schedule of the hospital in the morning and evening; however, they wanted to visit frequently. A 40-year-old parent said:

*“I would be happy if I had a chance to visit my child three times a day. Here, I am not farming, I am not keeping cattle. It is good if they [healthcare providers] allow me to visit my child and wife frequently as far as I am here to support them [the mother and the child]”*.[P13]

Some parents have also complained that the services given to their neonates at health facilities were usually delayed. This delay might cause the neonates to develop birth asphyxia, meconium aspiration syndrome, and other birth-related complications. For instance, a parent said:

*“We go to the health center in the evening and stay there up to midnight. Then, the night duty midwife goes, and another provider handover my wife. While the fluid was flowing from her uterus [premature membrane raptured], there was no sign of labor for several hours. She was only shouting [ehe, ehe, ehe…] without downward pushing. The next day, early in the morning, something happened either the fetus was breathless or sucking amniotic fluid. Then, they refer us to this hospital”.[P14]*

In addition, the shortage of space to take a rest is mentioned as a barrier for parents during their stay in the hospital. One parent described this as: "*I have faced a physical problem since I do not get a proper space to take a rest or take a nap within these two weeks”.[P5]*

Furthermore, parents complained that staying in the hospital was longer than their expectations. Although they had expected that they may return to their home within few days, they stayed more than a week in the hospital. This incident increased their expenses and put them in financial trouble.

##### Health workers related factor

Even though there were supportive HCPs, some parents reported their feelings that some HCPs were uncommitted, lacked discipline, and were uncooperative' to support them.

As parents explained, laboratory investigations were not done as a result of the absence of healthcare providers. For example, one parent explained as: “*X-ray was ordered but the experts were absent for the whole day and I was disappointed as a result of their absence”.* [P2]

Some parents explained that HCPs do not respect parents. A parent described as: “*The HCPs have several gaps. For instance, they cannot understand others’ problems, and they are negligent so that my baby is harmed. During labour, the midwife annoys her [my wife] and said do not shout. They [HCPs] stay a long time on social media. I am very upset since my baby has been injured although he could be born healthy. They do not consider us human; they undermined and forced us to go out, but I do not say these are the problems of all health professionals*”.[P14]

#### Theme V: maternal and child health factors

The severity of child health illnesses such as poor sucking reflex of the infant has contributed to parental stress in NICU. A parent said: *“…the child cannot take the expressed breast milk. I have a strong need and effort to save my child, but when I see the child, he is weak and has no improvement”. [P10]*

Another parent explained as *"…what can I do mister, my babies are serious ill. Doctors try to help them [twins] but they are not improved from their illness and I am too worried by their illness”. [P9]*

In NICU, most of the time, parents worry about the status of their neonates. However, in some instances when both the mother and the neonate get severely ill, the father experiences high stress and gives more attention to the mother than the neonate.

#### Theme VI: current occasions

The context of current pandemic infection [COVID-19] and seasonal situation (farming season) influenced parental stay in the hospital.

A parent explained: *“Because of the current problem [COVID-19] HCPs in the NICU do not give a positive response. They [HCPs] are not happy to give their professional support as expected from them”.* [P5]

A few parents explained that the neonatal admission time during farming season influences their stay in NICU. A parent said: *"The time now is June, which is the most important period for us as a farmer since it is our farming season. So, staying here for me is so challenging."[P2]*

## Discussion

This study was conducted to explore the experiences of parents in the NICU. Major identified themes were socio-economic, health facility-related, parents-health care providers' communication, maternal and child health-related factors, psycho-emotional factors, and current occasions.

Financial constraints were one of the parental challenges presented in the NICU. These challenges include a long stay in the hospital, extra costs for buying stock-out medicines, and their daily expenses like transportation and food. Similarly, reports showed that parents were challenged by additional costs during their NICU stay [30]. Other evidence showed that the financial burden on parents with babies admitted to a neonatal unit was high. The average cost per week is one-fourth of the total weekly income and includes lost income and additional expenses [44]. This agreement might be linked with the cost of long-time hospitalisation and other expenses outside of the NICU.

Parents complained that travelling a long distance from home to health facilities affected their stay and frequency of visits in NICU. Consistent with this study, parents faced hardships associated with travelling long distances from their homes to the health facilities [30]. Travel time influenced the frequency of visits, with fewer visits from those living furthest from the NICU. However, does not affect the content of parental visiting: a controlled prospective study [45]. This might be related to inaccessible NICU settings for parents nearby.

Anxiety, stress, worry, and confusion were the common psychological problems that parents experienced in the NICU. Not only the parents but also the whole family disturbed *and their families were too much confused”.* Comparably, the anxiety level of parents was high and had unforgettable moments [46]. Most parents were depressed and stressed due to the NICU atmosphere [7,47,48]. The more likely explanation of this similarity could be due to unfamiliar parents with NICU medical equipment.

In this study, sadness, crying and lack of self-control were emotional problems that were felt by parents in the NICU. Another study revealed that parents felt broken heartedness, disappointment, and fear. They perceived that “oxygen was bad because oxygen kills children”*;* it can be caused fears and worries for parents [26]. Furthermore, the NICU fathers expressed that the situation was out of their control [27,31]. Others also found that the most stressful events for parents were attached medical equipment and continuous noise of alarms in the NICU [33].

Even though parents wanted to visit their child frequently, the hospital had a limited visiting schedule. In line with this finding, reports showed that parents were challenged by strict visiting hours [30,49]. The service provided in NICU for neonates was delayed which might increase neonatal morbidity and mortality. Regarding the facility resources, shortage of space and sanitary situations was often overwhelming. Similarly, other studies showed that there were no private family rooms and a lack of waiting areas [8,30].

This study indicated that lack of compassionate and respectful care and unsupportive HCPs had negative consequences on parents that mean the HCPs were not disciplined, and they lack commitment and cooperation while they gave care in NICU. Other studies also showed that HCPs were unsupportive, careless, and negligent [7,8].

Although parents had no clear role and have limited engagement to care for the child, the interaction between HCPs and parents was good and supportive. Providing holistic information on the progress of the neonates’ health status for the parents had a positive impact on reducing their stress. This enhances parental service satisfaction in NICU. Other reports also substantiated that parents had gotten updated information about their infants’ health condition and their engagement was also optimal [8,17,26,30,31,49–52]. In contrast, other studies reported that parents were not receiving adequate information from HCPs about their babies’ progress [30,47].

Similarly, the poor medical condition of the neonates contributed to aggravating parental stress. This situation made them worried if the life of their child ends up with complications and death due to poor medical progress. This finding showed that the progress of infants’ medical condition was influenced by parental feelings [7].

In the current situation, parents were concerned about the quality of the care given to their children as a result of HCP frustrates COVID-19 transmission. Other evidence showed that during the COVID-19 pandemic occasion, parents experienced higher stress than the usual time. COVID-19 contagion also harmed parent-infant relationships [53]. This may be due to hospital restrictions having a significantly limited parental presence for NICU admitted infants [54].

Interviews were conducted in the NICU ward, the study participants might hide their feeling since they may be frustrated compromising the care given to their children. This study was also limited to include the health care providers' perspective, particularly in the parent-provider interaction.

## Conclusions and recommendations

Parents whose infants admitted to the NICU have faced both psychological and emotional problems such as, anxiety, stress, worry, hopelessness, confusion, anger, crying, sadness, frustration, dissatisfaction, happiness, regret, compliant, disappointment, bad feeling, self-blaming, nervousness, disturbance, and lack of self-control. In addition, parents were suffered from a lack of money, low professional support, and lack of space to take a nap. Furthermore, unavailability of some medicines, shortage of water for toilet and hygiene, lack of spaces to take rest, and limited time to visit their neonates were concerns for many parents. Likewise, parents reported their feelings that some HCPs had a lack of commitment, lacked discipline, uncooperative, and unsupportive to them. The poor medical condition of mothers and neonates and the current COVID-19 situation have threatened parents and their families in terms of getting optimal care in the NICU.

Based on the study findings, we recommended that accessibility of NICU services should be scaled up into the primary hospitals and health centres to avoid unnecessary expenditure of money and travelling a long distance from their home. The health team staff should provide ongoing education for the parents to minimise the risk of developing both psychological and emotional-related stress. The HCPs in NICU should involve parents to improve the care given to the neonate and alleviate negative emotions. The hospital should provide training on compassionate and respectful care for healthcare providers to enhance parent-healthcare provider communication and supports.

The supply chain management system of the health facilities should be strengthened to avoid a shortage of medicines. Sufficient waiting areas and space for the care of the neonates also should get due emphasis. The NICU setting should be designed in a way that HCPs and parents can manage their hygiene.

In addition, counselling services should be designed to promote the psychological health of NICU parents. Increase the access to personal protective equipment to prevent the transmission of contagious pandemic disease (COVID-19) in the hospital. Finally, further studies with longitudinal and qualitative design should be considered.

## Data Availability

The data that support the findings of this study are available on request from the corresponding author [DA]. The data are not publicly available due to their containing information that could compromise the privacy of research participants.
